# Prevalence and Genetic Characterization of Porcine Respiratory Coronavirus in Korean Pig Farms

**DOI:** 10.3390/ani14111698

**Published:** 2024-06-05

**Authors:** Ju-Han Kim, Jonghyun Park, Dong-Kyu Lee, Won-Il Kim, Young S. Lyoo, Choi-Kyu Park, Hye-Ryung Kim

**Affiliations:** 1 College of Veterinary Medicine, Konkuk University, Seoul 05029, Republic of Korea; jhkim2@sj.co.kr (J.-H.K.); lyoo@konkuk.ac.kr (Y.S.L.); 2 Swine Medical Corporation, Sunjin Bridge Lab, Icheon 17332, Republic of Korea; 3 Institute for Veterinary Biomedical Science, College of Veterinary Medicine, Kyungpook National University, Daegu 41566, Republic of Korea; parkjh@knu.ac.kr (J.P.); mooninlake@dodram.co.kr (D.-K.L.); parkck@knu.ac.kr (C.-K.P.); 4 DIVA Bio Incorporation, Daegu 41519, Republic of Korea; 5 College of Veterinary Medicine, Jeonbuk National University, Iksan 54596, Republic of Korea; kwi0621@jbnu.ac.kr

**Keywords:** porcine respiratory coronavirus, prevalence, genetic analysis, pig farm, Republic of Korea

## Abstract

**Simple Summary:**

The current epidemiology of porcine respiratory coronavirus (PRCV) in domestic pig farms in the Republic of Korea is not well understood. In this study, PRCV was found to still be present in Korean pig herds with a high seroprevalence. Genetic and phylogenetic analyses of PRCV S gene sequences suggested that Korean PRCV originated from European PRCV and has evolved in Korea. These findings will help expand knowledge about the epidemiology and genetic characteristics of PRCV in Korea.

**Abstract:**

Porcine respiratory coronavirus (PRCV) is a member of the species *Alphacoronavirus 1* within the genus *Alphacoronavirus* of the family *Coronaviridae*. A few studies have been conducted on the prevalence of PRCV since its first identification in 1997, but there have been no recent studies on the prevalence and genetic characterization of the virus in Korea. In this study, the seroprevalence of PRCV was determined in Korean pig farms using a commercially available TGEV/PRCV differential enzyme-linked immunosorbent assay kit. The farm-level seroprevalence of PRCV was determined to be 68.6% (48/70), similar to previous reports in Korea, suggesting that PRCV is still circulating in Korean pig herds nationwide. Among the 20 PRCV-seropositive farms tested in this study, PRCV RNAs were detected in 17 oral fluid samples (28.3%) from nine farms (45.0%), while TGEV RNAs were not detected in any sample. To investigate the genetic characteristics of Korean PRCV strains, genetic and phylogenetic analyses were conducted on PRCV spike gene sequences obtained in this study. The three Korean PRCV strains (KPRCV2401, KPRCV2402, and KPRCV2403) shared 98.5–100% homology with each other and 96.2–96.6% and 91.6–94.5% homology with European and American strains, respectively. A 224-amino acid deletion was found in the S gene of both Korean and European PRCVs but not in that of American PRCVs, suggesting a European origin for Korean PRCVs. Phylogenetic analysis showed that Korean PRCVs are more closely related to European PRCVs than American PRCVs but clustered apart from both, suggesting that Korean PRCV has evolved independently since its emergence in Korean PRCVs. The results of this study will help expand knowledge on the epidemiology and molecular biology of PRCV currently circulating in Korea.

## 1. Introduction

Coronaviruses (CoVs) are enveloped and pleomorphic RNA viruses that can infect a variety of animal hosts and are associated with a wide spectrum of clinical diseases, including enteric, respiratory, and neurological diseases [1,2]. Currently, six different CoVs from three genera of the family *Coronaviridae* are known to infect pigs: transmissible gastroenteritis virus (TGEV), porcine respiratory coronavirus (PRCV), porcine epidemic diarrhea virus (PEDV), and swine acute diarrhea syndrome coronavirus (SADS-CoV), also known as swine enteric alphacoronavirus (SeACoV) in the genus *Alphacoronavirus*; porcine hemagglutinating encephalomyelitis virus (PHEV) in the genus *Betacoronavirus*; and porcine deltacoronavirus (PDCoV) in the genus *Deltacoronavirus* [3,4]. TGEV, PEDV, PDCoV, and SADS-CoV are associated with enteric disease, PHEV is associated with neurological disease, and PRCV is mainly associated with respiratory disease in pigs [1,5].

PRCV is currently classified as *Alphacoronavirus 1* in the genus *Alphacoronavirus* of the family *Coronaviridae* [2]. The virus contains an enveloped, single-stranded, positive-sense RNA genome with a size of 28 kb. This genome encodes the following open reading frames (ORFs): ORF1a, ORF1b, spike (S), ORF3a, ORF3b, envelope (E) protein, membrane (M) protein, nucleocapsid (N) protein, and non-structural protein 7 (NS7) located between the 5′ and 3′ untranslated regions (UTRs) [5,6]. Among the structural proteins of PRCV, the S protein, a type I glycoprotein and receptor-binding protein, plays an important role in viral entry and interactions with hosts, which determines host range and tissue tropism [7,8].

PRCV was first identified in Belgium in 1984 and was confirmed to be a spike-gene-deletion mutant of TGEV [8]. As a variant of TGEV, the genome of PRCV is similar to that of the parental TGEV but is distinguished by a large deletion in the spike gene. The mutation in the S gene altered its tissue tropism and virulence, causing PRCV to infect respiratory tracts, unlike TGEV, which infects enteric organs [7,9]. The clinical impact of PRCV on pigs is not significant since the virus mainly infects the upper and lower respiratory tracts, leading to mild or sub-clinical respiratory disease in affected pigs [8,10]. Due to the highly transmissible nature of the respiratory virus, PRCV has rapidly spread to pig populations worldwide, resulting in a decrease in TGEV outbreaks due to cross-protective immunity between PRCV and TGEV [3,7,11].

In Korea, PRCV infection was first described in 1997 [12]. Subsequent serological studies using enzyme-linked immunosorbent assay (ELISA) that can distinguish between PRCV and TGEV infection showed that PRCV was widely distributed in Korean pig farms, with a seroprevalence at the farm level ranging from 61.3% to 78.4% [13,14,15]. Similar to European countries [7], TGEV outbreaks have declined after the emergence and nationwide distribution of PRCV in Korea [16,17]. According to the Korean Animal Health Integrated System (KAHIS, https://home.kahis.go.kr, accessed on 30 March 2024), no TGEV outbreaks have been reported in Korea since 2018. As TGEV outbreaks have not been reported in Korea for several years, the TGEV vaccine has rarely been used in Korean pig farms. Despite the rare use of TGEV vaccines, the current absence of TGE outbreaks in Korea is believed to be because PRCV is still prevalent and provides sufficient protective immunity to prevent TGE outbreaks in Korean pig herds. However, no research on PRCV has been conducted since the last seroprevalence study in 2009 in Korea [15] and updated epidemiological information on PRCV in Korean pig farms is lacking. The aims of this study were (1) to investigate the current seroprevalence of PRCV in Korean pig farms, (2) to determine the infection status of PRCV in PRCV-seropositive farms, and (3) to genetically characterize the PRCV strains currently circulating in Korea. The findings of this study can provide epidemiological and molecular biological insights into PRCV currently circulating in Korean pig farms.

## 2. Materials and Methods

### 2.1. Selection of Pig Farms and Sample Collection

To determine the seroprevalence of PRCV, five serum samples from finishing pigs were collected from each of 70 pig farms located in nine provinces in Korea. The 350 serum samples collected were stored at −20 °C until further analysis. To investigate the infection status of PRCV, oral fluid (OF) samples were collected from 20 pig farms that were confirmed to be seropositive for PRCV. Based on a previous report indicating that PRCV infection occurs in weaned pigs at 5–8 weeks of age [7], OF samples were collected from three pens housing 4- to 10-week-old pigs previously described by rope-based sampling method [18,19]. Briefly, animal interaction with the rope was limited to 20 min in each pen. After 20 min, the rope saturated with OF was removed from the pen, sealed in an airtight bag, and directly transported to the laboratory in a cool box with ice packs to minimize sample deterioration. Upon arrival at the laboratory, the OF samples were collected into 15 mL tubes and centrifuged at 3000 rpm for 10 min at 4 °C. Aliquots of each OF sample were placed in several 2 mL microcentrifuge tubes and stored at −70 °C until further molecular screening for PRCV.

### 2.2. ELISA for Serological Screening

A commercially available TGEV/PRCV differential blocking ELISA kit (INgezim Corona Diferencial 2.0, Ingenasa, Madrid, Spain) with high diagnostic sensitivity and specificity was used for the differential detection of antibodies against PRCV and TGEV [20]. The ELISA results were expressed as optical density and interpreted as positive, negative, or inconclusive for TGEV and PRCV following the manufacturer’s instructions. Based on the results, the seroprevalence of PRCV was determined at both the farm level and individual pig level. A farm was considered seropositive when at least one of the tested pigs was positive.

### 2.3. Nucleic Acid Extraction

Nucleic acids were extracted from 200 µL of each tested sample (OF, nasal swab, or rectal swab) using a TANBead nucleic acid extraction kit with a fully automated magnetic bead operating platform (Taiwan Advanced Nanotech Inc., Taoyuan, Taiwan). The extract was eluted with 100 µL elution buffer according to the manufacturer’s instructions and stored at −70 °C until use.

### 2.4. RT-qPCR Assays for Detection of PRCV and TGEV

Two reverse transcription quantitative polymerase chain reaction (RT-qPCR) assays were used for the differential detection of PRCV and TGEV. First, to detect both PRCV and TGEV, a previously described RT-qPCR assay using PRCV/TGEV N gene-specific primers and probe (PT-RT-qPCR) was carried out with RNA samples extracted from all OF samples collected in this study [21]. Subsequently, to specifically detect TGEV, another previously described RT-qPCR assay using TGEV S gene-specific primers and probe (T-RT-qPCR) was conducted with RNA samples that tested positive for both PRCV and TGEV [22]. The primers and probe used in the T-RT-qPCR were designed to target a region of the S gene sequence that is conserved in all TGEV strains but absent in closely related PRCV strains, enabling the specific detection of the TGEV S gene only [22]. Information on the primers and probes for the RT-qPCR assays is provided in Table S1. The RT-qPCR reactions were performed using a commercial one-step RT-qPCR kit (RealHelix^TM^ qRT-PCR kit [v4], NanoHelix, Daejeon, Republic of Korea) and a CFX96 Touch^TM^ Real-Time PCR Detection System (Bio-Rad, Hercules, CA, USA) according to the manufacturer’s instructions, as previously described [21,22]. Samples that were positive in both PT-RT-qPCR and T-RT-qPCR assays were considered TGEV-positive, whereas samples that were positive only in PT-RT-qPCR were considered PRCV-positive.

### 2.5. Sequencing of PRCV S Gene

To genetically characterize the PRCVs detected in this study, complete S gene sequences were obtained from three PRCV-positive samples with low Ct values. Complementary DNA (cDNA) fragments of two parts for the S gene were synthesized using a PrimeScript™ 1st strand cDNA Synthesis Kit (Takara Korea Biomedical Inc., Seoul, Republic of Korea). Sequence-dependent single-PCR amplifications were performed with two pairs of primers (S1F/S1R and S2F/S2R, Table S1) using Takara Ex Taq (Takara Korea Biomedical Inc., Seoul, Republic of Korea) following the manufacturer’s instructions. The amplified PCR products were purified using a commercial kit (GeneAll Expin™ Combo GP 200 miniprep kit, GeneAll, Seoul, Republic of Korea). Library preparation, library quality control, and sequencing were performed using the BITseq next-generation sequencing (NGS) service provided by a commercial company (BIONICS, Daejeon, Republic of Korea).

### 2.6. Genetic and Phylogenetic Analyses

The complete S gene sequences of 15 PRCV strains from various countries and a TGEV reference strain (Purdue, GenBank accession number DQ811789) were retrieved from the GenBank database (www.ncbi.nlm.nih.gov/genebank, accessed on 15 April 2024), as shown in Table S2. The S gene nucleotide (nt) and amino acid (aa) sequences of three Korean PRCV strains (KPRCV2401, KPRCV2402, and KPRCV2403) were aligned with the corresponding sequences of the 15 PRCV strains and a TGEV reference strain using MAFFT multiple sequence alignment software (version v7.490) [23]. Based on the MAFFT alignment, pairwise nt and aa sequence identities were determined using Geneious Prime (https://www.geneious.com, accessed on 15 April 2024). For the phylogenetic analysis, the IQ-TREE 2 software package (http://www.iqtree.org, accessed on 15 April 2024) was used [24]. The best-fit substitution model (GTR + F + G4) was selected using ModelFinder (https://www.mathworks.com/help/simulink/slref/modelfinder.html, accessed on 15 April 2024) [25], and a maximum likelihood phylogenetic tree was constructed through ultrafast bootstrap analysis with 1000 replicates [26]. The phylogenetic tree was visualized using the iTOL phylogenetic tree viewer [27].

## 3. Results

### 3.1. Serological Prevalence of PRCV in Korean Pig Farms

**Figure 1:**
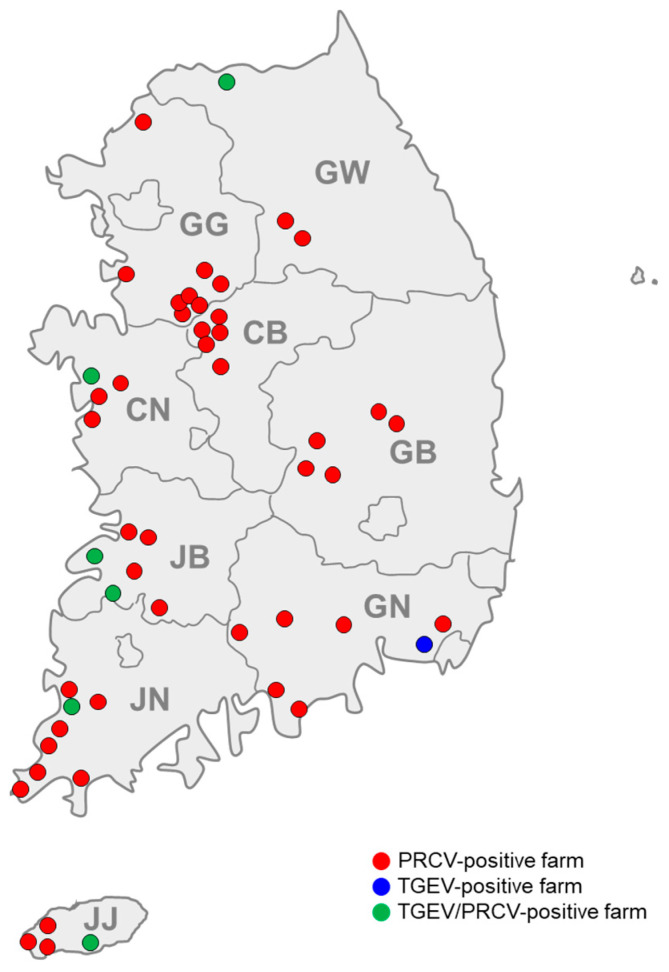
Regional distribution of seropositive pig farms for porcine respiratory coronavirus (PRCV) and/or transmissible gastroenteritis virus (TGEV) in Korea. Red circles (●), blue circles (●), and green circles (●) indicate PRCV antibody-positive, TGEV antibody-positive, and both TGEV and PRCV antibody-positive farms, respectively. The abbreviations GW, GG, CB, CN, GB, GN, JB, JN, and JJ on the map correspond to Gangwon-do, Gyeonggi-do, Chungcheongbuk-do, Chungcheongnam-do, Gyeongsangbuk-do, Gyeongsangnam-do, Jeollabuk-do, Jeollanam-do, and Jeju-do, respectively.

The seroprevalence of PRCV in Korean pig farms, as determined in this study, is shown in Table 1. The pig-level seroprevalence of PRCV and that of TGEV were determined to be 41.1% (144/350) and 4.3% (15/350), respectively. A total of 3 out of 350 serum samples (0.9%) were determined to be inconclusive (doubtful for specific antibodies to TGEV), while the remaining 188 out of 350 sera (53.7%) were negative for porcine coronavirus (PRCV and TGEV). Of the 70 farms tested, 49 (70.0%) were seropositive for porcine coronavirus (PRCV and/or TGEV), including 42 PRCV antibody-positive (60.0%), one TGEV antibody-positive (1.4%), and six TGEV/PRCV antibody-positive farms (8.6%). The overall farm-level seroprevalence of PRCV and that of TGEV, including the farms positive for both, were determined to be 68.6% (48/70) and 10.0% (7/70), respectively. Geographically, PRCV-seropositive farms were found throughout all nine provinces, while TGEV-seropositive farms were limited to six provinces in Korea (Table 2 and Figure 1). Among the seven TGEV antibody-positive farms, one (AJ farm) located in Gyeongsangnam-do province was positive for TGEV antibody only, while the remaining six were also positive for PRCV antibody and were located in five different provinces (KS farms in Gangwon-do, SW farm in Chungcheongnam-do, AS and JY farms in Jeollabuk-do, and ITS farm in Jeju-do provinces) (Table 3 and Figure 1).

### 3.2. Detection and Prevalence of PRCV in Seropositive Pig Farms

Seventeen OF samples from nine farms were found to be PRCV/TGEV-positive using the PT-RT-qPCR assay, but no positive results were obtained with the T-RT-qPCR assay, indicating that the positive results were due to singular infection of PRCV (Table 4). Based on these results, the viral prevalence of PRCV was determined to be 45.0% (9/20) at the farm level and 28.3% (17/60) at the sample level. Among the PRCV-positive OF samples, three samples (two from the same farm and one from another farm) with lower Ct values (higher viral loads) were selected for the sequence analysis of the PRCV S gene.

### 3.3. Analysis of S Gene Sequences of Korean PRCV Strains

**Figure 2:**
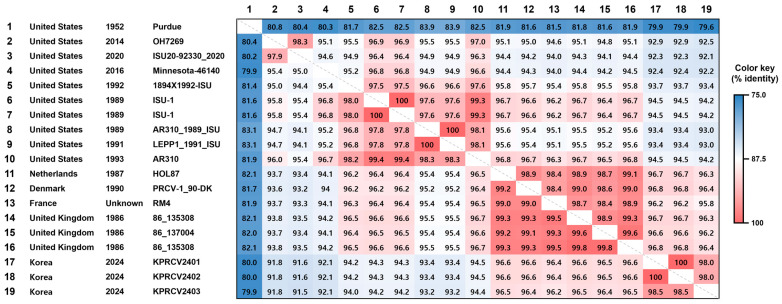
The pairwise identity matrix based on spike genes of 18 porcine respiratory coronavirus (PRCV) strains and a transmissible gastroenteritis virus (TGEV) reference strain. The matrix illustrates the similarity of nucleotide (nt) and amino acid (aa) sequences between strains based on pairwise comparisons with the spike (S) gene sequences of 18 PRCV strains and a TGEV reference strain. The percent similarities of nt and aa sequences between strains are shown in the lower and upper triangles of the matrix, respectively. The pairwise nt and aa identities of the 19 S gene sequences are color-coded based on the color key provided on the right side of the figure. The TGEV Purdue strain was used as an outgroup.

**Figure 3:**
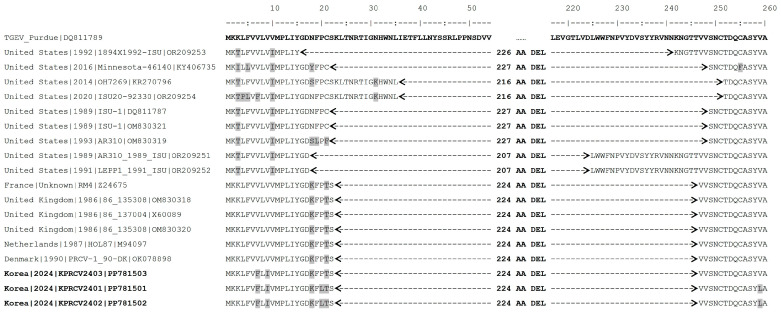
Sequence alignment of the N-terminal region of spike genes of 18 porcine respiratory coronavirus (PRCV) strains and a transmissible gastroenteritis virus (TGEV) reference strain. The amino acid (aa) sequence of the TGEV Purdue reference strain is shown in the first line, followed by the sequences of 18 PRCV strains. Sequence numbers are based on the position of aa residues in the S protein sequence of the TGEV Purdue strain. Compared to the sequence of the TGEV Purdue strain, the changed aa residues in the PRCV strains are highlighted in gray. The start and end of deletion regions in the sequences of each PRCV strain are indicated with left-pointing and right-pointing arrows, respectively. The length of the aa deletion region for each PRCV strain is indicated in the middle of the corresponding sequence. The strain names of the three Korean PRCVs are written in bold font.

In this study, three complete S gene sequences (3678 nucleotides in length) were obtained from PRCV-positive OF samples collected from two pig farms. Two sequences (KPRCV2401 and KPRCV2402) were obtained from the same pig farm (Farm ID SPR), while the third sequence (KPRCV2403) was obtained from a different pig farm (Farm ID, OK). Alignment of the three Korean PRCV S gene sequences showed that two sequences (KPRCV2401 and KPRCV2402 strains) from the same SPR farm shared 100% nucleotide identity, but they shared 98.5% nucleotide identity with the sequence of the KPRCV2403 strain from the OK farm (Figure 2). The complete S gene sequences of the Korean PRCV strains were compared with corresponding sequences of 15 PRCVs, including 6 European and 9 American strains (Table S2). Based on the complete S gene sequences, the six European strains shared 99.0–99.8% nt or 98.4–99.6% aa homology, and the nine American strains shared 94.1–100.0% nt or 94.6–100.0% aa homology. The three Korean PRCVs shared 96.2–96.6% nt or 95.8–96.8% aa homology with the European strains and 91.6–94.5% nt or 92.1–94.5% aa homology with the American strains (Figure 2). Among the six European PRCV strains, the PRCV-1_90-DK strain from Denmark in 1990 and the 86_135308 strain from the United Kingdom in 1986 were the most closely related to the Korean PRCV strains with 96.6% nt and 96.9% aa homology. To further analyze the deletion pattern of Korean PRCV strains, the aa sequences of the N-terminal region of the S gene were compared with those of 15 previously reported European and American PRCV strains, as well as a reference TGEV Purdue strain (Figure 3). Compared to the reference TGEV Purdue strain, a 224-amino acid deletion at residues from 23 to 246 was identified in the S genes of all Korean PRCVs, which was consistent with European PRCV strains but differed from American PRCV strains, which showed deletions of 207, 216, 226, or 227 amino acids (Figure 3).

### 3.4. Phylogenetic Analysis Based on the Complete S Gene Sequences of PRCV

**Figure 4:**
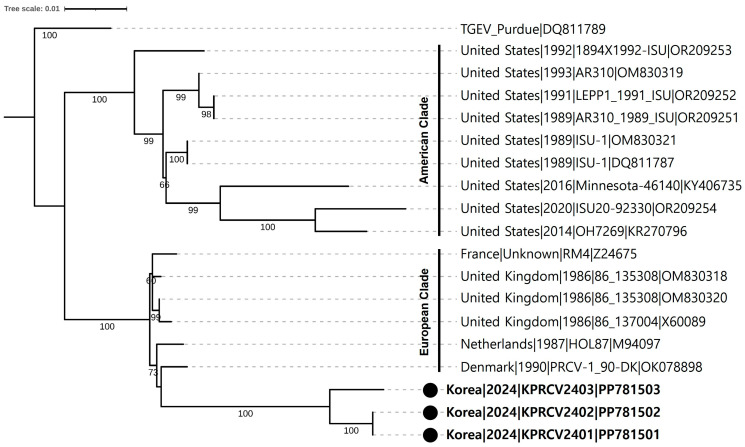
Phylogenetic tree of global porcine respiratory coronavirus (PRCV) strains based on the complete spike gene sequences. Black circles indicate three Korean PRCV strains sequenced in this study. The PRCV strains were grouped into two clades: American and European clades. Detailed information about each strain is provided on the right side of the tree. The numbers in each branch represent bootstrap values greater than 50% based on 1000 replicates. Scale bars indicate nucleotide substitutions per site. The transmissible gastroenteritis virus Purdue strain was used as an outgroup.

For the phylogenetic analysis of Korean PRCV strains, a phylogenetic tree was constructed using S gene sequences of 15 non-Korean PRCV strains and a TGEV reference Purdue strain (Table S2). In the phylogenetic tree, 18 PRCV strains were classified into two clades, European and American clades, distinct from the TGEV outgroup (Figure 4). A clear geographical grouping pattern was observed in the constructed phylogenetic tree. Six previously reported European PRCVs were grouped into the European clade, and nine previously reported American PRCVs were grouped into the American clade. However, the three Korean PRCV strains were separated from all European PRCVs with high bootstrap values, although they were grouped into the European clade (Figure 4).

## 4. Discussion

The worldwide distribution of PRCV has been considered to play a critical role in the decline of TGEV infection in the global swine industry, including Korea [3,7,11]. Although PRCV is still believed to be circulating in swine herds, recent information on the epidemiology and molecular biology of the virus is limited in Korea, where the last seroprevalence study was conducted in 2009 [15]. In this study, we investigated the seroprevalence of PRCV and TGEV in Korean pig herds using a commercial ELISA kit that can differentiate between PRCV and TGEV antibodies. The seroprevalence of PRCV was determined to be 41.1% (144/350) at the pig level and 68.6% at the farm level (48/70) (Table 1). In previous Korean studies, the pig-level seroprevalence of PRCV was reported to be 53.1% in the late 1990s [13], 46.5% in 2006 [9], and 63.7% in 2009 [15], which were slightly higher than the findings of this study. The farm-level seroprevalence was reported as 61.3% in the late 1990s [13] and 78.4% in 2006 [14] in previous Korean studies, which were similar to the results of this study. Despite some differences in seroprevalence between this and previous studies, it can be confirmed that PRCV has been consistently circulating in Korean pig herds with high prevalence from the late 1990s to the present.

On the other hand, anti-TGEV antibodies were detected in 15 sera collected from 7 pig farms, indicating 4.3% of pig-level seroprevalence and 10.0% of farm-level seroprevalence in this study (Table 1 and Table 3). The pig-level seroprevalence of TGEV determined in this study was similar to those in previous Korean studies [13,15]. These results suggest that TGEV may still be circulating in some Korean pig herds despite the high seroprevalence of PRCV. However, direct evidence for the presence of TGEV in these seven pig farms could not be found due to the lack of suitable enteric samples for TGEV detection in this study. In this regard, it is important to note recent Chinese studies indicating that TGEV is still prevalent in Chinese pig herds, particularly in certain provinces of China [28,29]. Therefore, further extensive surveillance studies are needed to confirm the presence of TGEV and determine its exact prevalence in Korean pig herds.

Virological surveillance of PRCV has been based on individual animal specimens, including nasal swabs and lung tissue samples [21,30,31]. However, aggregate specimens, such as OF samples, have not been used to survey PRCV despite their potential advantages over individual animal specimens [19,32,33]. In this study, 28.3% (17/60) of OF samples from 45% (9/20) of pig farms were found to be PRCV-positive using RT-qPCR assays (Table 4). Recent studies in the US and Spain have reported varying detection rates of PRCV. In a study in the US, PRCV was detected in only 0.4% (5/1245) of lung homogenates from respiratory-diseased pigs on farms across the country [34]. Based on the results, the authors suggested that PRCV may no longer be widespread in US pig herds and that factors other than PRCV may be contributing to the decline of TGEV outbreaks, though these factors were not specified. In contrast, a recent study in Spain detected PRCV at a much higher rate, with 48% of nasal swab samples from respiratory-diseased weaned pigs testing positive [31], consistent with the results from this study using OF samples. The reason for the particularly low detection rate of PRCV in the US remains unknown. To address this, an international collaborative study is needed to investigate PRCV surveillance in global swine herds in the future.

Global PRCVs were classified into two genetic groups based on geographic distribution: European and American groups. Each PRCV group has been proposed to be derived from a different origin [7,35]. Despite the high seroprevalence of PRCV in Korea, there has been no study on the genetic characterization of PRCV circulating in Korea [13,14,15]. In this study, three complete S genes of Korean PRCVs were sequenced and compared. The sequences of KPRCV2401 and KPRCV2402 strains derived from the same farm were 100% identical to each other but differed from the sequence of the KPRCV2403 strain derived from a different farm with 98.5% identity (Figure 2), suggesting that genetically different PRCV strains coexist in Korea. Sequence alignment between the S gene sequences of Korean and non-Korean PRCVs revealed that Korean PRCVs were genetically more similar to European strains than to American strains (Figure 2). Further analysis of the S gene deletion pattern revealed that both Korean and European PRCVs had the same deletion pattern, whereas American PRCVs had different deletion patterns (Figure 3), as previously described [7,35]. Phylogenetic analysis showed that Korean PRCVs were clustered separately from European PRCVs within the European clade (Figure 4). These results suggested that Korean PRCV was originally derived from European PRCVs and has evolved independently after being introduced into Korea. Such independent evolution of Asian PRCVs has already been reported in a Japanese study [36]. To the best of our knowledge, this is the first report on the genetic characteristics of Korean PRCV. However, this study has a limitation in that only three PRCV sequences from two Korean pig farms were analyzed. Further studies are needed to secure more genetic sequences of Korean PRCVs and to elucidate the genetic diversity of the PRCVs distributed in Korea.

Since the emergence of PRCV, several studies have been conducted to evaluate the pathogenicity of this virus in pigs. The pathogenicity of the virus has been shown to vary from non-pathogenic to low-pathogenic depending on the viral strain used in the study [36,37,38,39,40]. However, despite the low virulence of PRCV, this virus may be involved in the occurrence of porcine respiratory disease complex (PRDC) in the fields. Previous studies have shown that co-infection with PRCV and common respiratory pathogens, such as porcine reproductive and respiratory syndrome virus, swine influenza virus, and *Bordetella bronchiseptica*, can cause more severe clinical signs and growth retardation compared to a single infection of each pathogen [7,40,41,42]. Considering the high prevalence of PRCV identified in this study and the circulation of various traditional and novel respiratory pathogens in Korean pig farms [43,44,45,46], it is likely that co-infection between PRCV and other respiratory pathogens occurs frequently in the fields, which may contribute to worsening clinical outcomes of PRDC. Therefore, further studies are needed to elucidate the role of PRCV in the pathogenesis of PRDC in the fields.

## 5. Conclusions

In this study, we confirmed that PRCV is still circulating in Korean pig herds with a high prevalence, similar to previous reports in Korea, which is believed to contribute to suppressing outbreaks of TGEV in Korean pig herds. Genetic and phylogenetic analyses showed that Korean PRCV strains are more closely related to European strains than American strains but cluster separately from previously reported European and American PRCV strains, suggesting that Korean PRCV has evolved independently since its emergence in Korea. Based on the high prevalence of PRCV identified in this study, continuous monitoring and surveillance are needed to elucidate its role in the pathogenesis of PRDC in Korea. The findings of this study will enhance our understanding of the epidemiology and molecular biology of PRCV currently circulating in Korea.

## Figures and Tables

**Table 1:** Seroprevalence of PRCV and TGEV determined through ELISA in this study.

Seroprevalence at the Pig-Level			Seroprevalence at the Farm-Level		
Interpretation	No. of Positive	Positive Rate (%)	Interpretation	No. of Positive	Positive Rate (%)
PRCV-positive	144	41.1	PRCV-positive	42	60.0
TGEV-positive	15	4.3	TGEV-positive	1	1.4
Inconclusive	3	0.9	P/T-positive ^a^	6	8.6
Negative	188	53.7	Negative	21	30.0
Total	350	100.0	Total	70	100.0

^a^ P/T-positive indicates that both porcine respiratory coronavirus (PRCV) and transmissible gastroenteritis virus (TGEV) antibodies were detected in serum samples from the same farm using the TGEV/PRCV differential enzyme-linked immunosorbent assay (ELISA) kit.

**Table 2:** Regional distribution of pig farms based on ELISA results.

Province	No. of Tested Farm	No. of Farm Categorized by ELISA Results (%) ^a^			
		PRCV	TGEV	PRCV/TGEV	Negative
Gangwon-do	4	2	-	1	1
Gyeonggi-do	9	8	-	-	1
Chungcheongbuk-do	8	5	-	-	3
Chungcheongnam-do	8	3	-	1	4
Gyeongsangbuk-do	6	4	-	-	2
Gyeongsangnam-do	8	6	1	-	1
Jeollabuk-do	7	4	-	2	1
Jeollanam-do	12	7	-	1	4
Jeju-do	8	3	-	1	4
Total	70 (100.0)	42 (60.0)	1 (1.4)	6 (8.6)	21 (30.0)

^a^ Based on the results of the transmissible gastroenteritis virus (TGEV) and porcine respiratory coronavirus (PRCV) differential blocking enzyme-linked immunosorbent assay (ELISA) used in this study, each pig farm was categorized as PRCV-positive, TGEV-positive, PRCV/TGEV-positive, or -negative.

**Table 3:** ELISA results for seven TGEV antibody-positive pig farms.

Farm ID	Province ^a^	No. of Tested	Antibody Detection by ELISA ^b^			
			TGEV	PRCV	Inconclusive	Negative
KS	GW	5	3	2	-	-
SW	CN	5	2	3	-	-
AJ	GN	5	2	-	-	3
AS	JB	5	2	2	1	-
JY	JB	5	3	2	-	-
ITS	JN	5	2	1	-	2
GB	JJ	5	1	2	-	2
Total		35	15	12	1	7

^a^ GW, CN, GN, JB, JN, and JJ represent Gangwon-do, Chungcheongnam-do, Gyeongsangnam-do, Jeollabuk-do, Jeollanam-do, and Jeju-do provinces, respectively. ^b^ According to the instructions provided with the transmissible gastroenteritis virus (TGEV) and porcine respiratory coronavirus (PRCV) differential blocking enzyme-linked immunosorbent assay (ELISA) used in this study, the assay results for each serum sample were interpreted as TGEV antibody-positive, PRCV antibody-positive, inconclusive, or TGEV/PRCV antibody-negative.

**Table 4:** Detection of porcine respiratory coronavirus (PRCV) in oral fluid samples collected from PRCV-seropositive pig farms.

Method ^a^	Detection at the Farm Level			Detection at the Sample Level		
	No. of Tested	No. of Positive	Detection Rate (%)	No. of Tested	No. of Positive	Detection Rate (%)
PT-RT-qPCR	20	9	45.0	60	17	28.3
T-RT-qPCR	20	0	0	60	0	0

^a^ PT-RT-qPCR, a reverse transcription followed by quantitative polymerase chain reaction (RT-qPCR) assay that can amplify both PRCV and TGEV N genes; T-RT-qPCR, an RT-qPCR assay that can specifically amplify only the TGEV S gene and cannot amplify the PRCV S gene.

## Data Availability

The complete S gene sequences of Korean PRCVs obtained in this study were submitted to the GenBank database (accession number: PP781501, PP781502, and PP781503).

## Supplementary Materials

The following supporting information can be downloaded at: https://www.mdpi.com/article/10.3390/ani14111698/s1, Table S1: Primers and probes used to detect porcine respiratory coronavirus (PRCV) and transmissible gastroenteritis virus (TGEV). Table S2: Porcine respiratory coronavirus (PRCV) and transmissible gastroenteritis virus (TGEV) strains analyzed in this study.
