# Supplementary material for: Prevalence and Genetic Characterization of Porcine Respiratory Coronavirus in Korean Pig Farms
Source: Animals (Basel). 2024 Jun 5;14(11):1698. doi: 10.3390/ani14111698 (PMC11171391; doi:10.3390/ani14111698)
Supplement: Supplementary file 1 [file animals-14-01698-s001.zip › animals-3034371-supplementary.pdf]

Supplementary Table S1. Primers and probes used to detect porcine respiratory coronavirus (PRCV) and transmissible gastroenteritis virus (TGEV).

Method [a]	Primer/probe	Sequence (5′–3′) [b]	Reference
PT-RT-qPCR	NF	AGCTATTGGACTTCAAAGGAAGATG	[21]
	NR	CATAGGCATTAATCTGCTGAAGGAA	
	NP	FAM-TCACGTTCACACACAAATACCACTTGCCA-BHQ1	
T-RT-qPCR	TGE-SF	TCTGCTGAAGGTGCTATTATATGC	[22]
	TGE-SR	CCACAATTTGCCTCTGAATTAGAAG	
	TGE-SP	FAM-**Y**TAAGGGCTCACCACCTACTACCACCA-BHQ1	
Sequencing for S gene	S1F	CTCCTAAATTCAAGTGTCGTTG	In this study
	S1R	ACTGGTGTAGTGTAAACCTGA	
	S2F	GTGAACCTGTCATAACCTATTC	
	S2R	TAAATTTAATGGACGTGCACTT	

[a] PT-RT-qPCR, a reverse transcription followed by quantitative polymerase chain reaction (RT-qPCR) assay that can amplify both PRCV and TGEV N genes; T-RT-qPCR, an RT-qPCR that can specifically amplify only the TGEV S gene and cannot amplify the PRCV S gene.
[b] Bold text in the sequences of the TGE-SP probe indicates a degenerate base: Y, C, or T. FAM, 6-carboxyfluorescein; BHQ1, Black Hole Quencher 1.

Supplementary Table S2. Porcine respiratory coronavirus (PRCV) and transmissible gastroenteritis virus (TGEV) strains analyzed in this study

No.	Strain	Location	Collected year	GenBank accession no.	S gene length (nt / aa)
1	86_135308	United Kingdom	1986	OM830318	3,678 / 1,226
2	86_137004	United Kingdom	1986	X60089	3,678 / 1,226
3	86_137008	United Kingdom	1986	OM830320	3,678 / 1,226
4	HOL87	Netherlands	1987	M94097	3,678 / 1,226
5	PRCV-1_90-DK	Denmark	1990	OK078898	3,678 / 1,226
6	RM4	France	Unknown	Z24675	3,678 / 1,226
7	Minnesota-46140	United States	2016	KY406735	3,666 / 1,222
8	AR310	United States	1993	OM830319	3,669 / 1,223
9	AR310_1989_ISU	United States	1989	OR209251	3,729 / 1,243
10	LEPP1_1991_ISU	United States	1991	OR209252	3,729 / 1,243
11	1894X1992-ISU	United States	1992	OR209253	3,672 / 1,224
12	OH7269	United States	2014	KR270796	3,699 / 1,233
13	ISU-1	United States	1989	DQ811787	3,669 / 1,223
14	ISU20-92330	United States	2020	OR209254	3,669 / 1,233
15	ISU-1	United States	1989	OM830321	3,669 / 1,223
16	KPRCV2401	South Korea	2024	PP781501	3,678 / 1,226
17	KPRCV2402	South Korea	2024	PP781502	3,678 / 1,226
18	KPRCV2403	South Korea	2024	PP781503	3,678 / 1,226
19	TGEV Purdue	United States	1952	DQ811789	4,344 / 1,448
